# Valedictory Address

**Published:** 1875-04

**Authors:** Thomas S Latimer

**Affiliations:** Prof. of Anatomy.


AMERICAN JOURNAL

OF

DENTAL SCIENCE.

Vol. VIII. THIRD SERIES—APRIL, 1875. No. 12.

### ARTICLE I.

### *Valedictory Address*

BY THOMAS S LATIMER, M. D., PROF. OF ANATOMY.

**Delivered at the Thirty-Fifth Annual Commencement of the Baltimore College of Dental Surgery.**

It is I believe the uniform custom in all colleges at the close of the collegiate year, publicly to present to the graduating class the diplomas which testify the efficient discharge of their duties as pupils, and, in schools teaching special branches of science as a vocation, assert their qualification properly to discharge the duties it involves.

In accordance with this time honored custom we are met this evening, and the duty has devolved on me to speak the few words of advice and encouragement usual on these occasions. In casting about for a theme of interest, I could think of none better than the familiar one, trite and commonplace enough to all those who have passed this period of their lives, but I believe always of living interest to the graduating class. I remember with what profound attention I, at the same period of my life, listened to the advice then spoken, though I can not now suppose it was at all novel, nor characterized by any especial eloquence.

I trust therefore in attempting plainly and simply to point out to you the nature and purpose of your life work, and the principles by which you should be guided in its execution, I may not altogether fail to interest you. I do not mean to speak to you as dentists of the work belonging to your especial duties, but as men of that which concerns you in common with all other men.

It has been said that no man is truly happy until his pleasure grows out of his work, as naturally as does a color petal grow out of a flower. This no doubt is a saying difficult for you to understand, and for years to come more difficult for you to feel. It is however a great truth which you must sooner or later learn if your happiness is ever to be established on a sure foundation. The many alluring phantasms of pleasure which in early life we so eagerly pursue, are all transitory and in dying dower us with a "harvest of barren regrets." But work, real true work, into which a man's heart and soul enters is its own exceeding great reward, but it must be pure in its purpose as well as strong in its strife, and with divine affections bold to follow wherever it shall lead. Many of you, perhaps most of you, have chosen your vocation with a view simply to making an honest livelihood, not recognizing in life any nobler purpose than the attainment of worldly success. There is however, true work and false work, heroic work and unheroic work for every man alive whatever his vocation. It behooves us then at the outset of our careers, to find what is the true and heroic, and to seek it, and it only, thenceforward. I know none of you require to be told of the unfruitfulness of idleness; of that as practical men in a work, a day world you are well assured. In idleness alone there is despair; but in him who actively and earnestly works, though mammon were his God, there is hope; but alas! if this be the God of your idolatry, there is only hope

An existence of play sustained by the blood of other creatures, as such an existence needs must be, answers very well for gnats and sucking fish, but not for men; neither days nor lives can be made holy by doing nothing in them. Man is born on a battle field and it is appointed unto him to wrestle not to reign, and it is well with him if after toil and strife he may reach a purer air. It is with all things worthy to be had, as it is with the metal gold; nature might have placed it upon the surface of the earth where all who sought might find it with ease; but she chose rather to bury it deeply in the bowels of the mountain, where you must dig long and painfully to find any, and may dig long and painfully and find none. Yet it is the work that blesses man with increase of strength, rather than the gold to which he attains, for "God in cursing gives us better gifts than men in benediction," and God says sweat for brows; thus then can we understand the wise injunction: "Get work, get work, be sure 'tis better than what you work to get."

Mr. Ruskin has truly and beautifully said, "Every man has a true and a false life. His true life is like that of lower organic beings, the independent force by which he moulds and governs eternal things. It is a force of assimilation by which he converts everything around him into food, or into instruments; and which however humbly or obediently it may follow the guidance of a superior intelligence, never forfeits its own authority as a judging principle, as a will capable of either obeying or rebelling. His false life is indeed but one of the conditions of death or stupor but it acts even when it cannot be said even to animate, and is not always easily known from the true. It is that life of custom or accident, in which many of us pass much of our time in the world; that life in which we do what we have not purposed, and speak what we do not mean, and assent to what we do not understand; that life which is overlaid by the weight of things external to it, and is moulded by them instead of assimilating them; that which instead of growing and blossoming under any wholesome dew, is crystalized over with it as with hoar frost, and becomes to the true life what an arborescence is to the tree, a candid agglomeration of thoughts and habits foreign to it, brittle, obstinate, and icy, which can neither bend nor grow, but must be crushed and broken into bits if it stands in our way. All men are liable to be in some degree frost bitten in this sort; all are partly encumbered and crusted over with idle matter, only if they have real life within them they are always breaking this bark away in noble rents until it becomes like the black strips upon the birch tree, a witness of their own inward strength." Though all men have not that inward strength which enables them to break through the environments of time and circumstance, and mould them to their will, yet all have power for striving, and though they may not wear the laurel, they may feel that they have contributed something, though but a widow's mite, to the widening of men's thoughts.

"At least not rotting like a weed,
But having sown some glorious seed,
Fruitful of further thought and deed."

With the old monks it used to be said—*laborare est orare*—to labor is to worship—and it is a saying worthy of all acceptance. It is indeed the only worship wherein God manifests himself. Prayer and praise and thanksgiving are well, but they are only means to an end. Means by which our lives are framed on principles of active benevolence, that we may go about *doing* good, not dreaming it all day long. Nursing "dainty sympathies in some delicious ease" is well if that they finally grow into fruit of generous action. An old Mahomedan maxim commends itself very strongly to my judgment—"One hour spent in the execution of justice is worth a thousand years of prayer." Thought and sentiment are well, but only in so far as they prompt to action, for the life of man is properly an action, and not a thought though it were the noblest. All *true* thought is convertible into action; it is indeed the test of its truth that it is thus convertible. The life of all Gods figures itself to us as an earnestness of infinite battle ; battle for the true and good against the false and evil. Everywhere in nature is ceaseless activity ; there is a mill wheel for the tiniest rivulet to turn ; but not the less may it break into dimples and laugh in the sun, than when it dances over the sandy shallows. Destiny has indeed no other way of moulding us into shape and use. Set even a formless chaos rolling and a world is born. He then who has found his true work, his life purpose, has found the true source of all blessedness, of all hopefulness, of all knowledge. "The knowledge that will hold good in working cleave thou to that, for nature herself accredits that, says yea to that. Properly thou hast no other knowledge but what thou has got by working ; the rest is all a hypothesis of knowledge ; a thing to be argued of in schools, a thing floating in the clouds in endless logic vortices, till we try it and fix it. Doubt of whatever kind can be ended by action alone."

But the question recurs to us, what is true work, and what is false work, and how are we to elect in a world where so much is thrust upon us in which we have no choice ?

The first great aim in every man's life should be to determine his capabilities. To ascertain in what particulars, and to what extent he is qualified to work, and then to do it with all his might, and from other motives than that of gain. To learn the nature and extent of our capabilities is no doubt the most difficult thing we can find to do. Carlyle in his pungent way says, alas ! our young son is all budding with capabilities, and we see not yet which is the main and true one. Always too the new man is in a new time, under new conditions ; his course can be the *fac simile* of no prior one, but is by its nature original. * * * Thus in a whole embroglio of capabilities, we go stupidly groping about to find which is ours, and often clutch the wrong one ; in this mad work must several years of our small term be spent, until the poor blind youth by practice, acquire notions of distance, and becomes a seeing man. Nay, many so spend their whole term, and in ever new expectation, ever new disappointment, shift from enterprise to enterprise; till at length as exasperated striplings, of three score and ten, they shift into their last enterprise, that of gettine buried."

It is perhaps well for us that rigorous task master Hunger thrusts us into some form of employment before we have settled this question. For indeed it may be doubted whether any solution can be had save in and through labor. It will not do to ponder too long what we are qualified to do, but rather experimentally test that which is nearest to hand. Thus will vague wavering capability soonest become converted into fixed indubitable performance which is indeed the " mirror where in our soul sees its natural lineaments, for it is only by what we have done, that we come to know of what we are capable.

I do not counsel you to despise the getting of money; it is helpful and needful in all life whatever its aims and tendencies. Put money in your purse is an injunction to be by no means despised, yet the true wealth of a man is the number cf things which he loves and blesses, which he is loved and blessed by. Every good man, it has been well said, holds his life in his hand ready to give it for a noble purpose, not to sell it. Too much time is necessary to accomplish any useful purpose to spend much of it in counting the profits to accrue from it. We get no good by being ungenerous, even to a book, and counting so much profit from so much labor; it is only when we gloriously forget ourselves and plunge headlong into its pages enamored of the salt and savor of life in it that we attain to the essential good it contains.

The element of love then necessarily enters into all true work, and just in proportion as that love is pure and unselfish is the work born of it good and pure and enduring. In the earlier days ot our lives even the best workers love the ends of work better than the work itself. Wealth and power and ambition are the potential motives that prompt to action, sustaining and encouraging us in the early drudgery that belongs to the alphabet of labor. A. B. C. were nothing but drudgery if it did not open vistas of volumed wealth that are to reward that early toil. Nor are these in themselves unworthy motives. If the wealth is sought for benificent ends; the power to suppress the wrong and uphold the true; the pride of place be that the place whereon we stand, be it a heaven kissing hill or a lowland valley, be the place where justice may be done and prayer and thanksgiving may ascend, then are these motives worthy in themselves, and the work growing out of them has in it an element of truth, but if real work is to be done, work wherein our hands shall not tire nor our hearts grow weary, we must superadd to these, love of the particular work itself. The sculptor must love sculpturing more than the fame or wealth accruing, however worthily both may be made to subserve noble ends; the painter must love the creations of essential beauty more than the award of the academy, before marble shall breath beneath his chisel or canvas glow with the inspiration of his genius.

It is physically impossible for any well educated, warm hearted and brave man to make money the chief end of his being. All men love to make money and properly. Every good soldier likes to receive his pay, and the best of them will grumble not a little if it is withheld, but the chief purpose of his life is to win battles, not to receive pay. If pay is the end, slavery is the means, it matters not whether we are bought with praise or gold. The distinguishing sign of slavery is, to have a price and be bought for it; the distinguishing sign of heroism is to have an aim and if needs be, to die for it. In the pursuit of truth, sorrow and privation must be borne; but what need he care who "stands upon some lofty mountain thought and feels his spirit stretch into a view" that round him are icy rocks. His soul is singing at a work apart behind the walls of sense, and truth is given him for sole vision, let contending tempests blow never so sharply about his naked head.

True work then is work that we love; it is the fashioning of our souls into whatsoever may be the product of our minds and hands, expressing in it our highest capabilities in the direction in which we have sought to engage our energies, and that equally, whether it be in the plugging of a tooth, or in the redemption of a land. If that which we do is that for which we are especially fitted, and in doing it we have made no reservation of our powers, we have attained the limit of heroism in the daily avocations of life. "Act well your part, there all the honor lies." It matters not what the part may be, the honor lies not in it, but in doing it well. Therefore "produce, produce though it should be the pittifullest infinitesimal fraction of a product, produce it in God's name."

There can be no disguising the fact that much of weariness and heartsoreness enters into labor; that there are times when even "the labor we delight in" will not "physic pain;" but alas! into all life does heartsoreness enter, yet busy brains and active hands, erect against it the most formidable barriers.

While something of worldly prosperity is unquestionably essential to the happiness of all ordinarily constituted mortals, yet it is equally true, that if out of the rugged block of adversity you have carved nothing but a stout statue of prosperity to set up among your household gods it will never lay happy hands upon your head in benediction. No man shall or should barber the true and beautiful for barley feeding and material ease, and be happier therefore. If the gratification of your pride be the object of your labors, it will defeat its own object. Do what your hands find to do reverently, because all perfect work presupposes reverence. Enthusiastically because God within us, is the only way to get God within it; and, if you esteem the work you are set to do as a sacrafice of your lives, remember that it is only when sacrifice is offered with "liberal joy" that it is acceptable to God or a theme of praise among men.

Every man worthily striving fails not of here and there a little success to keep him in heart; a kindly spoken well done to cheer him ere he has learned to dispense with cheer, but it comes more surely if it has not been sought as an end. Yet he who strives most earnestly, with the purest heart and strongest brain, must ever find " an infinite void twixt the absolute aim and the partial achievement." Checks and disasters grow in the veins of action highest reared; * * * and every action which has gone before where of we have record, trial did draw bias and thwart, not answering the aim, nor that unbodied figure of the thought which gave it surmised shape." Yet no true man falters for these reasons: What though the arrows of his aim are blunted, and the needs of his trust are broken, still must he gird himself anew for the strife, accounting it nobler to have fallen in front rank and be overtrodden by the abject rear, than to have done and hang like a rusty mail in monumental mockery. Who is it that would not rather, like the roman general, wear his " honor owing wounds" hid 'neath his mantle, than expose them in the market place, and for that he wears them, receive the most sweet voices that declare him consul? If such there are among you let him never be a seeker after truth. The quest is not for him. If prompted by a passing impulse, he should vow a vow that for a twelve month and a day he will follow truth, his vow shall not save him from following wandering fires.

The quantity of work done and forgotten, writes Mr. Carlyle, that lies silently under our feet in this world, that escorts and attend us, supports and keeps us alive wheresoever I stand or walk, whatsoever I think or do gives rise to reflection. Is it not enough to strike the thing called fame into total silence for a wise man. Scarcely two hundred years back can fame recollect articulately at all; and there she but maunders and mumbles, she manages to recollect a Shakespeare or so, and prates considerably like a goose about him." It is indeed only here and there from all past time that we encounter a name at whose mention " oblivion shrinks like a thing reproved," and if any among you shall give such loose rein to his fancy as to dream of being one among the least of these, let him remember that not only is the mighty genius needed but also the martyr's spirit ere he can be accounted worthy to share their "grand adversity." Let me advise you then, whatever the aim or purpose of your lives, not to

> "Count too much on human praise
> That comes to guerdon after days."

Perhaps the sublimest picture in human nature, is that of the philosopher Kepler, rejoicing at the discovery of a truth, when after seventeen years of struggle, "full conviction burst upon his mind; he had won the goal, the struggle of seventeen years ended, God was vindicated, and the philosopher in the wild excitement of his glorious triumph exclaims:

"Nothing holds me. I will indulge my sacred fury. If you forgive me I rejoice; if you are angry I can bear it. The die is cast. The book is written, to be read either now or by posterity; I care not which. It may well wait a century for a reader, since God has waited six thousand years for an observer."

Intellectual work, what we call study, needs no praisers, it is itself a satisfaction of all wants. It is wealth in poverty, liberty in bondage, health in sickness, society in solitude. It consoles sorrow, assuages pain, brings gladness to eyes that fail with wakefulness and tears, and ache for the dark house and long sleep. "Surely, says McCauley," "it is no exaggeration to say that no external advantage is to be compared with that purification of the intellectual eye, which gives us to contemplate the infinite wealth of the mental world; all the hoarded treasures of its primeval dynasties, all the shapeless ore of its yet unexplored mines."

But in addition to the satisfaction of our material wants, the gratification of ambition, and the love we ourselves have for the work we have set ourselves to do; there are two other essential elements in all perfect work. 1st. That no pitiful envy of another's success should be possible to us.

Not only because remembering that "emulation hath a thousand sons," and that we must often be outstripped in the race for truth and lose the honor of its discovery; and that if we admit a thing so blind, embrace it as our natural good, it must prove a serpent to sting us from our peace; but because you have not, and can not have that perfect love for the object of your pursuit which alone entitles you to succeed, if you are not glad to see it attained more closely even by your most formidable rival. It is not an august inward impulse which prompts your action if the superior success of another rankles in your bosom. It has been beautifully and truthfully said, by Mrs. Browning, when considering Cimabue, contemplating the superior merit of the shepherd boy, Giotto's, sketches:—

"I hold, too,
That Cimabue smiled upon the lad,
At the first stroke that passed what he could do,
Or else his virgins smile had never had
Such sweetness in it. All great men who foreknew
Their heirs in art, for arts sake have been glad,
And bent their old white heads as if uncrowned,
Fanatics of their pure ideals still
Far more than of their triumphs."

Great success in anything, the smallest, is not possible for littleness, and envy is the brood of little minds.

There is room enough in this fair world for the weakest man in it to live and die, as well as for all the strongest to live well, and strive their own way by their individual heat, seeking their own ideals.—

"The ultimate perfection leaning bright
From out the sun and stars to bless the leal
And earnest search of all for fair and right."

The last important element in the character of true workers to which I would call your attention, especially if their labors lead them in the direction of investigation rather than in the application of that which is known to the daily uses of life, is a divine courage to accept and boldly to acknowledge that which reason has determined to be true. The earnest seeker has naught to do with likes and dislikes. He has no right to hesitate to accept aught because his emotional nature says no to it. What he has to do first of all is to divest himself as entirely as is possible for a human being of all pre-conceptions, and to examine each fact or theory upon the examination of which he may be engaged, by the pure white light of reason, and when reason says yea to it, there is nothing authorized to say nay. "This is a repulsive, a revolting doctrine, I will not acccept it" is too often upon the lips of avowed seekers after truth. It matters not how revolting, with that you have absolutely nothing to do. The sole question for you to ask is, is it true? and if the answer be yea, and you hesitate to accept it because it is not what you would like to be true, you are recreant to all virtue. Nor would I counsel a too hasty acceptance of alleged facts:

> "Ran haste is half-sister to delay."

> "Hold fast the good, define it well,
> For fear divine philosophy
> Should push beyond her mark, and be
> Procuress to the lords of hell."

Yet whilst holding fast the good, do not ever suppose it separable from the true, nor hesitate to loose that hold the moment reason and judgment shall say, "that which thou holdest for truth is not truth, but error." It is a base abandonment of mind to resign our right of thought, and a baseness which meets the penalty due to baseness. I know of no sadder sight under heaven than to see a strong man holding to his heart the delusion once dear to him as a living truth, but now incapable of giving or receiving warmth. Nor can any truth long remain obnoxious to the zealous lover and worker of good. Free thought and bold are alone the instrumentalities by which the obscure can be made plain, and the darkness be converted into light. A thinking man, if his heart be pure, is the greatest enemy the prince of darkness has to encounter. It pre-supposes and includes a brave heart. The motto inscribed upon his banner is, truth though the heavens should crush me for following it; no error though a world should account it good.

Honorable industry then sums up the aim and end of a good man's life. All earnestness and industry will be useless, unless they are consecrated by your resolutions to be in all things men of honor; not honor in the common sense only, but in the highest. * * * Though it be not exacted of you, yet exact it of yourselves, this vow of stainless truth. Your hearts are, if you leave them unstirred, as a tomb in which a God lies buried. "Vow yourselves crusaders to redeem that sacred sepulchre;" remembering that in all things, "your strength shall be as the strength of ten," if that "your hearts are pure."

Yet I would not figure life to you as altogether a continuous strife, though it be in the acquisition of knowledge, the determination of truth, the dissemination of good, the purification of self. Rest and play, mere rest and play are constituent parts of a life well spent. Though work be our highest and most enduring pleasure, it need not be our sole pleasure. "The full face of the soul turned hopefully and steadily toward the true, must catch across its ridge the idealized sunlight of the beautiful." Having tilled the soil and sown the seed, we may stretch our limbs at noon and "Tranquil *muse* upon tranquility."

While the warm wooings of the sunny day
Tremble along the frame and harmonize
The attempered brain, that even the saddest thoughts
Mix with some sweet sensations, like harsh tunes
Played deftly on a soft toned instrument.

The sun should be our type of a great worker; its high office to guide the earth through the heavens and warm it into fruition; yet it does not disdain to "add a color to the rose," to throw a perfume o'er the violet," to enchant the winds to music 'till the listening spirit walks—

"Exempt from mortal care
God-like o'er the clear billows of sweet sound."

If it never swerves from the path of duty, nor admits a stain upon its spotless crest, it counts it no shame to lie like a loved locket upon the bosom of a quiet lake, "and make its silvery splendor pant with bliss."

Moving straight on it rejoiceth like a strong man as it goes. Work and play, alike perfect after their kinds, each unassoiled and unassoilable. Make it your model; work as it does zealously and purely; rejoice as it does afar from "The purple lined palace of sweet sin," and your days shall be full of peace.

And now it only remains for me to say good bye! trusting that so much prosperity may be accorded to each of you, as is consistent with the most perfect development of your highest capabilities, and no more; to leave with you in the name of the Faculty of the College, the benediction of your *alma mater*, and for myself to thank you for your patient attention.

